# Support for sexual and reproductive health and rights in Sub-Saharan Africa: a new index based on World Values Survey data

**DOI:** 10.1186/s12978-024-01820-2

**Published:** 2024-06-25

**Authors:** Signe Svallfors, Karin Båge, Anna Mia Ekström, Yadeta Dessie, Yohannes Dibaba Wado, Mariam Fagbemi, Elin C. Larsson, Helena Litorp, Bi Puranen, Jesper Sundewall, Olalekan A. Uthman, Anna E. Kågesten

**Affiliations:** 1 Department of Sociology, Stanford University, 450 Jane Stanford Way, Stanford, CA 94305-2047 USA; 2 Department of Global Public Health, Karolinska Institutet, Stockholm, 171 77 Sweden; 3 Venhälsan, Department of Infectious Diseases, Södersjukhuset, Stockholm, 118 83 Sweden; 4 Department of Clinical Science and Education, Södersjukhuset, Stockholm, 118 83 Sweden; 5 School of Public Health, College of Health and Medical Sciences, Haramaya University, Harar, P.O. Box 235, Ethiopia; 6 African Population and Health Research Center, Nairobi, Kenya; 7 Kantar Public, 376 Ikorodu Road, Lagos, Nigeria; 8 Yucca Consulting Limited, 16b Ogunsona Street, Lagos, Nigeria; 9 Department of Women’s and Children’s Health, Karolinska Institutet, Stockholm, 17177 Sweden; 10 Department of Women’s and Children’s Health, Uppsala University, Akademiska sjukhuset, Uppsala, 75185 Sweden; 11 World Values Survey Association, Institute for Future Studies, Stockholm, 10131 Sweden; 12 Social Medicine and Global Health, Lund University, Malmö, 214 28 Sweden; 13 HEARD, University of KwaZulu-Natal, Durban, South Africa; 14 Warwick Medical School, Warwick Centre for Global Health, University of Warwick, Coventry, UK

**Keywords:** Sexual and reproductive health and rights, Attitudes, Index, Factor analysis, Sub-Saharan Africa, World Values Survey

## Abstract

**Background:**

Addressing attitudes is central to achieving sexual and reproductive health and rights (SRHR) and Agenda 2030. We aimed to develop a comprehensive index to measure attitudinal support for SRHR, expanding opportunities for global trend analyses and tailored interventions.

**Methods:**

We designed a new module capturing attitudes towards different dimensions of SRHR, collected via the nationally representative World Values Survey in Ethiopia, Kenya, and Zimbabwe during 2020–2021 (*n* = 3,711). We used exploratory factor analysis of 58 items to identify sub-scales and an overall index. Adjusted regression models were used to evaluate the index according to sociodemographic characteristics, stratified by country and sex.

**Results:**

A 23-item, five-factor solution was identified and used to construct sub-indices reflecting support for: (1) sexual and reproductive rights, (2) neighborhood sexual safety, (3) gender-equitable relationships, (4) equitable masculinity norms, and (5) SRHR interventions. These five sub-indices performed well across countries and socioeconomic subgroups and were combined into a comprehensive “SRHR Support Index”, standardized on a 1–100 scale (mean = 39.19, SD = 15.27, Cronbach’s alpha = 0.80) with higher values indicating more support for SRHR. Mean values were highest in Kenya (45.48, SD = 16.78) followed by Ethiopia (40.2, SD = 13.63), and lowest in Zimbabwe (32.65, SD = 13.77), with no differences by sex. Higher education and being single were associated with more support, except in Ethiopia. Younger age and urban residence correlated with more support among males only.

**Conclusion:**

The SRHR Support Index has the potential to broaden SRHR attitude research from a comprehensive perspective – addressing the need for a common measure to track progress over time.

**Supplementary Information:**

The online version contains supplementary material available at 10.1186/s12978-024-01820-2.

## Plain language summary

Sexual and reproductive health and rights (SRHR) are becoming increasingly polarized worldwide, but researchers have previously not been able to fully measure what people think about SRHR. More research about this topic is needed to address discriminatory norms and advance SRHR for all. In this study, we added new questions to the World Values Survey collected in Ethiopia, Kenya, and Zimbabwe during 2020–2021. We used statistical methods to develop an index capturing to what extent individuals’ attitudes were supportive of SRHR. This index, which we call the SRHR Support Index, included 23 survey questions reflecting support for five related dimensions of SRHR. Those dimensions were (1) sexual and reproductive rights, (2) neighborhood sexual safety, (3) gender-equitable relationships, (4) equitable masculinity norms, and (5) SRHR interventions. We found that individuals in Kenya were more supportive of SRHR, followed by Ethiopia and then Zimbabwe. There were no differences in support of SRHR between men and women, but individuals who were single and those with higher education were more supportive of SRHR, except in Ethiopia. Younger men living in urban areas were also more supportive. Our SRHR Support Index enables researchers, policymakers, and others to measure attitudes to SRHR in countries across the world and over time, based on new data from the World Values Survey that are readily available online. If combined with other sources of data, researchers can also investigate how people’s support of SRHR is linked to, for example, health and policy.

**Supplementary Information:**

The online version contains supplementary material available at 10.1186/s12978-024-01820-2.

## Background

Sexual and reproductive health and rights (SRHR) were put at the forefront of international population policy in the mid-1990s when the United Nations conferences in Beijing and Cairo emphasized rights to bodily autonomy and women’s empowerment [1]. The importance of SRHR for human development was reaffirmed when Agenda 2030 was adopted by the United Nations General Assembly in 2015, primarily in Sustainable Development Goal (SDG) #3 on health and well-being and #5 on gender equality [2]. Despite important progress to ensure universal access to SRHR as part of these goals – reflected in reduced rates of maternal mortality, HIV incidence, and increased access to modern contraceptives globally [2], the global progress has been far from equal within and across countries, with growing resistance and backlash towards sexual and reproductive rights – such as abortion and the rights of sexual minorities in many contexts. Recent examples include the anti-SRHR declaration “Geneva Consensus Declaration” signed by 34 countries in 2020 (15 from sub-Saharan Africa (SSA)), the US Supreme Court’s decision to overturn Roe v. Wade in 2022, and the overall shrinking space for civil society organizations promoting SRHR [3–8].

Resistance or support towards SRHR is intrinsically linked with social norms, which may impact an individual’s capacity to make decisions about their own body and sexuality, freely express their gender and sexual identity, and decide when, if and with whom to form relationships, have sex, marry, and have children [2]. Such norms can be difficult to measure due to their multidimensional nature, leaving a large gap in our current understanding about views and opinions related to SRHR. Existing indices and scales have mainly focused on specific SRHR aspects such as women’s empowerment or gender norms using data from, for example, the Demographic and Health Surveys (DHS) or the Global Early Adolescent Study (GEAS) [9–12]. A global SRHR index has also been developed to track the US government’s commitment to SRHR in global health programs [13]. These previous measures were either developed to assess limited aspects of SRHR outcomes, policies, and funding streams [13] or were based on data collected among certain populations such as married women or adolescents [9–12]. Consequently, there is a need for new, comprehensive measures using nationally representative data regardless of sex, age, or relationship status, to tap into the intersecting dimensions of gender, power, and decision-making that underlie support for SRHR.

### Study aim

We aimed to develop a comprehensive index measuring individuals’ support for SRHR based on a novel module integrated into the nationally representative World Values Survey (WVS) data collected in Ethiopia, Kenya, and Zimbabwe; and to assess the validity of the index across sociodemographic characteristics. Findings can be used to facilitate future empirical research and guide the operationalization and prioritization of survey items, thereby broadening opportunities for global comparisons and trend analyses within and across countries, and over time.

### Conceptual framework

Our study is grounded in the 2018 Guttmacher-Lancet Commission integrated definition of SRHR, which builds on globally established human rights conventions and emphasizes the right for all individuals to enjoy a state of physical, emotional, psychological, and social well-being in relation to all aspects of sexuality and reproduction [2]. We used this framework both to guide the development of new items in the WVS questionnaire as well as the selection of existing WVS items in the analysis – aiming for a representation of different SRHR domains from a comprehensive perspective – and to contextualize and interpret our findings.

We also draw on social norm theory, where norms are defined as socially or culturally constructed informal rules about what is considered acceptable or appropriate when it comes to sexual and reproductive preferences, identities, choices, desires, roles, and relationships, as well as SRHR information and services in a given group, community or setting [2, 14–17]. In this paper, we focus on individuals’ attitudes towards common social and cultural perceptions and practices related to SRHR, which we refer to as “support for SRHR” [14].

## Methods

### Study design and setting

We used cross-sectional nationally representative data on individuals’ support for SRHR collected for the first time as part of the 7th global WVS wave. W﻿hile the WVS has been conducted in most countries in the world, data collection in SSA has remained limited. For the current study, we used data from a new WVS module on attitudes toward SRHR developed by our team, implemented between February 2020–June 2021 in three sub-Saharan African countries where such information has been less available: Ethiopia, Kenya, and Zimbabwe. Despite great progress to ensure SRHR for all over the past decades, these countries carry a prevailing high burden of adverse outcomes such as maternal mortality and morbidity, complications from unsafe abortion, gender-based violence, adolescent childbearing, and limited sexual rights (Supplementary Table A1) [18]. The three countries also differ in terms of their abortion legislation, prevalence of HIV, and harmful practices, as well as their population size, health, and political systems [19]. They are all signatories of key SRHR documents such as the Protocol to the African Charter on Human and People’s Rights on the Rights of Women in Africa [20], which provides a policy framework to ensure SRHR, including ending harmful practices and ensuring many, although not all, reproductive rights.

### Data source and participants

The WVS has collected data on sociocultural values and beliefs through standardized face-to-face interviews with representative population-based samples of adults since 1981, available open-access. An in-depth explanation of the WVS data collection procedures to minimize bias as well as a full methodological report for each country can be retrieved from https://www.worldvaluessurvey.org. For the present study, the full WVS sample in the three included countries comprised 3,711 males and females aged 18 years or above (Ethiopia *n *= 1,230, Kenya *n *= 1,266, Zimbabwe *n *= 1,215). Data were collected following WVS standards including mechanisms to ensure the safety of data via direct uploading and storage of data on a highly secure password protected server. No identifying data from the participants were collected, removing the requirement for a written consent form. However, all participants were requested to provide oral informed consent, witnessed by the interviewer. The research was conducted in compliance with the principles laid out in the Declaration of Helsinki. An ethical permit was granted from the Swedish Ethical Review Authority to analyze data that were collected abroad in Sweden (Dnr 2020–05314).

### Variables

Data used in the present study are based on a new SRHR module, which was first developed and piloted by our team in the Nigerian WVS wave 7 in 2018 [21]. The new module was further adapted and expanded with additional questions drawing on the Guttmacher-Lancet SRHR definition for the three countries in this study [19], which is why we did not include the Nigerian sample here. 

The standard WVS questionnaire includes 14 items covering some aspects of SRHR, such as women’s role in society, subjective health status, empowerment, life satisfaction, as well as attitudes to, e.g., homosexuality, abortion, premarital sex, and divorce. In the new module, we added 44 measures of attitudes related to different domains of SRHR as per the Guttmacher-Lancet Commission definition, including child marriage, early childbearing, comprehensive sexuality education, contraceptive use, skilled birth attendance, gender-equitable relationships and gender norms, premarital sex, infertility, abortion, and sexual and gender minority rights. Supplementary Table A2 presents an overview of the complete 58-item battery and their response options. Most questions asked respondents to indicate their agreement with statements on a Likert-type response scale, such as “Please tell me if you strongly agree, agree, neither agree nor disagree, disagree, or strongly disagree with the following statements: A man should always have the final say about decisions in his relationship or marriage.” Some questions asked: “How frequently do the following things occur in your neighborhood – very frequently, quite frequently, not frequently, or not at all? Sexual assault/rape.” Finally, a third set of questions asked, “Please tell me for each of the following actions whether you think it can always be justified, never be justified, or something in between, using this card: Abortion.” The latter set of items was based on a 10-graded scale. Details on the development of the new SRHR module have been described elsewhere [19, 21].

Beyond country, we also included five sociodemographic characteristics as covariates in the current analysis: age groups (18–24; 25–29; 30–39; 40–49; 50 +), sex (we use the terms male/men or female/women interchangeably), place of residence (urban; rural), highest educational level (primary or lower; secondary; tertiary), and relationship status (married or cohabiting; divorced, separated, or widowed; single).

### Patient and public involvement in the study

We used deidentified secondary data publicly available on the WVS website. Patients or the public were not involved in the design, conduct, reporting, or dissemination plans of our research.

### Statistical analysis

We began with a descriptive analysis, which indicated that 25% of the full sample (*N* = 927) were missing responses for up to 46 items in the SRHR module. Non-response on these items varied by country, relationship status, and education, but not as much by age, sex, or place of residence. We excluded respondents with less than a 25% response rate on the SRHR items, i.e., those who did not respond to 14 or more of the total 58 items (*N* = 45, < 2% of the original WVS sample). Non-response rates on sociodemographic variables were low (< 3%) and deemed unproblematic for our analysis. The initial analytical sample thus included 3,666 respondents (Ethiopia *n* = 1,223, Kenya *n* = 1,228, Zimbabwe *n* = 1,215).

While the survey items were developed using a deductive approach, to capture the comprehensive nature of the Guttmacher-Lancet definition of SRHR, we applied an inductive, data-driven approach to develop the actual index, rather than “forcing” items into specific domains. We did this by using Exploratory Factor Analysis (EFA) to identify the most parsimonious number of hypothetical dimensions that could explain covariation among the 58 included items. EFA is useful to identify the factor structure for a set of variables inductively, without constraining items to load on specific factors [22]. Bartlett’s test of sphericity and the Kaiser–Meyer–Olkin (KMO) measure of sampling adequacy showed that EFA was feasible.

All items were coded so that higher values indicate more supportive attitudes towards SRHR. For example, responses to the statement “Sexual education helps people make informed decisions” with response options 1 = agree completely, 4 = disagree completely, were subsequently reverse coded so that higher scores represented greater agreement, and thus more support for SRHR interventions [2]. We did not reverse code negative statements where disagreement indicated more support for SRHR, such as “A man shouldn't have to do household chores”.

Drawing on previous studies [23] we did an initial assessment of how many factors to retain based on Polychoric correlations (given the Likert-type variables), principal component analysis (PCA), scree plots, and parallel analysis (PA). Then, iterated principal factors (IPF) with oblique rotation were used to determine the appropriate number of factors.

Criteria for determining factor adequacy were established a priori: parsimony was preferred over complex loadings that were salient on more than one factor. We retained factors with a minimum of three coefficients loading > 0.40, an item uniqueness of < 0.70, and that were conceptually meaningful according to the Guttmacher definition. We used Cronbach’s α to test the internal consistency between factor items, with ≥ 0.7 considered acceptable reliability [21].

We tested the resulting factor solution with retained items: for the full pooled sample, each of the three countries, and on subsamples disaggregated by the five sociodemographic characteristics.

We started the EFA with complete cases (*n* = 2,722) on 58 variables. Results from the initial PCA and scree plots (Supplementary Figure A1) suggested a 9-factor solution, but some factors did not fulfill the criteria. We thus reduced the number of factors and excluded irrelevant items iteratively, until a solution was reached that met all the criteria outlined above. By excluding some items, the number of complete cases increased (*n* = 3,135).

The factor items were combined into subindices by extracting latent scores from each factor using regression scoring. Mean scores for the subindices were combined with equal weighting into an overall index by adding the scores and then dividing it by the number of subindices. The overall index and subindex scores were standardized to a 100-graded scale for interpretability. Higher scores represented more agreement with the achievement of sexual and reproductive health and rights.

We further conducted a sensitivity analysis to assess the proposed factor solution using multiple imputations based on 10 samples with standardized scales to fill in missing data on the SRHR items [22].

Finally, we conducted multivariable linear regression models to assess the association between the index scores with sociodemographic characteristics. Both pooled and stratified models by country and sex were conducted. This final step served both to test the construct validity of the index as a potential source of bias and as an empirical evaluation of characteristics associated with support for SRHR in the study settings.

In the regression models, we included only complete cases on the retained index items and the five sociodemographic variables (*n* = 3,113 or 84% of the original WVS sample). The covariates included in the regression models are displayed in Table 1. These sociodemographic characteristics did not differ notably from the original WVS sample (Supplementary Table A5). A sample flowchart is available in Supplementary Figure A3.

**Table 1:** Sociodemographic characteristics of the study sample (*n* = 3,113)

Variable	n	%
**Age**		
*18–24*	862	27.69
*25–29*	614	19.72
*30–39*	776	24.93
*40–49*	426	13.68
*50* +	435	13.97
**Sex**		
*Man*	1,595	51.24
*Woman*	1,518	48.76
**Place of residence**		
*Urban*	1,205	38.71
*Rural*	1,908	61.29
**Relationship status**		
*Married or cohabiting*	1,843	59.20
*Divorced, separated, or widowed*	313	10.05
*Single*	957	30.74
**Education**		
*Primary or lower*	1,512	48.57
*Secondary*	1,092	35.08
*Tertiary*	509	16.35
**Country**		
*Ethiopia*	945	30.36
*Kenya*	1,011	32.48
*Zimbabwe*	1,157	37.17
**TOTAL**	**3,113**	**100.00**

## Results

### Factor analysis results

Results from the EFA suggested a 23-item, five-factor solution (Figure A2 in Supplementary Material, *n* = 3,135). This solution was robust to using multiple imputation to fill missing data, using both PCA and maximum likelihood (ML) extractions, varimax and promax rotations, and excluding non-responses on SES variables. The solution performed well across the three countries, albeit with minor variations with some items having low loading (< 0.50) and high uniqueness (> 0.70) in each country. The factor solution was stable across countries, age, sex, education, residence, and relationship status (rotated factor loadings by country and sociodemographics are available upon request).

Table 2 shows the five identified factors, along with their respective rotated factor loadings, reflecting support for different aspects of SRHR. Supplementary Table A3 further displays loadings for each of the 23 variables across all five factors included in the SRHR Support Index. These factors correlated at less than 0.7, indicating low risk of multicollinearity (Supplementary Table A4).

**Table 2:** Exploratory factor analysis

SRHR dimension	WVS item	Variable	Loading
***FACTOR 1: Sexual and Reproductive Rights***			
Non-discrimination related to sexuality, sexual orientation, and gender identity	Q182	Justifiable: Homosexuality	0.86
Reproductive empowerment	Q184	Justifiable: Abortion	0.84
Consensual, non-violent relationships	Q183	Justifiable: Prostitution	0.88
	Q189	Justifiable: For a man to beat his wife	0.72
	Q185	Justifiable: Divorce	0.66
Satisfying sexual life	Q186	Justifiable: Sex before marriage	0.76
	Q193	Justifiable: Having casual sex	0.83
***FACTOR 2: Neighborhood Sexual Safety***			
Consensual, non-violent relationships	H311	How often in neighborhood: Sexual assault/rape	0.71
	H312	How often in neighborhood: Women and girls trading sex for money	0.83
	H313	How often in neighborhood: Men and boys hurting women and girls	0.92
	H314	How often in neighborhood: Men and boys making unwanted sexual comments or gestures toward girls or women	0.87
***FACTOR 3: Gender-Equitable Relationships***			
Gender-equitable relationships	H325	A man should always have the final say about decisions in his relationship or marriage	0.56
	H328	There is no doubt that gainful employment is good but that what most women really want is a home and children	0.73
	H329 (K)	On the whole, family life suffers when women work full time	0.58
	H330	It is a man's job to earn money and a woman's job to take care of home and family	0.67
Consensual, non-violent relationships	H326 (Z)	If a man has a girlfriend or wife, he should know where she is all the time	0.60
***FACTOR 4: Equitable Masculinity Norms***			
Gender-equitable relationships	H348	A man who talks a lot about his worries, fears, and problems doesn't reserve respect	0.54
	H350	A real man should have as many sexual partners as he can	0.79
	H351	A man should use violence, to get respect, if necessary	0.82
***FACTOR 5: SRHR Interventions***			
Reproductive empowerment	H337	Women should have access to safe abortion services to terminate an unwanted pregnancy	0.53
	H341 (E)	Contraceptives should be available for everyone, whether or not one is married	0.58
	H344	A couple who cannot conceive should have access to infertility services	0.60
SRH information	H343	Sexual education helps people make informed decisions	0.62

Variables with low loading (< 0.50) and high uniqueness (> 0.70) are marked (E) for Ethiopia, (K) for Kenya, and (Z) for Zimbabwe

The *first factor*, which we labeled “Sexual and Reproductive Rights”, included seven items measuring the justification of different rights related to one’s body, sexuality, sexual interactions, and intimate relationships (Cronbach’s α = 0.92). Three of these items are part of the previously validated “WVS Choice” subindex (abortion, divorce, and homosexuality) [24], which were now combined with an additional four items related to intimate partner violence, pre-marital sex, causal sex, and sex work. All the items in this factor share the same format; asking the extent to which the respondent believes a specific way of being or behaving can be justified on a 1–10 scale (higher values representing greater agreement), without contextualizing the behavior to a specific situation.

The *second factor* included four items tapping into the perceived exposure of women and girls to sexual harassment and violence by men and boys, as well as the perceived frequency of sexual assault/rape, and exchange of sex for money or goods, in the respondents’ neighborhood (Cronbach’s α = 0.86). These items all reflected women’s and girls’ freedom from, or risk of, sexual violence and exploitation within their local contexts [14–17]. We thus labeled this factor “Neighborhood Sexual Safety”.

The *third factor* was characterized by five items measuring perceptions related to male control and decision-making in relationships or marriage, gendered divisions of work in and outside the household, and power in intimate relationships (Cronbach’s α = 0.71). As these items all tap into the importance of ensuring gender equality in relationships as a domain of SRHR [2], we labeled it “Gender-Equitable Relationships”.

The *fourth factor* included three items drawn from the validated “Man Box scale” developed by Equimundo [25], capturing disagreement with masculinity norms promoting sexual prowess and violence, and that men should avoid talking about their feelings (Cronbach’s α = 0.65).Since all items were coded so that higher values indicated more support for SRHR, we labeled this factor “Equitable Masculinity Norms”.

Finally, the *fifth factor,* which we named “SRHR Interventions”, contained four items measuring perceptions related to different essential interventions for SRHR [2], including safe abortion services, contraception, infertility treatment, and sexuality education (Cronbach’s α = 0.57).

Next, we extracted each factor to scores resulting in five specific subindices, which were further combined into a full index. The resulting “SRHR Support Index” consisted of 23 items with an overall Cronbach’s α = 0.80, indicating high internal reliability. Factors 1–3 demonstrated good internal consistency, and while alpha scores were lower for Factors 4–5, they were deemed acceptable given their substantive relevance and contribution to overall reliability (which was lower without these factors) [22].

**Fig. 1:**
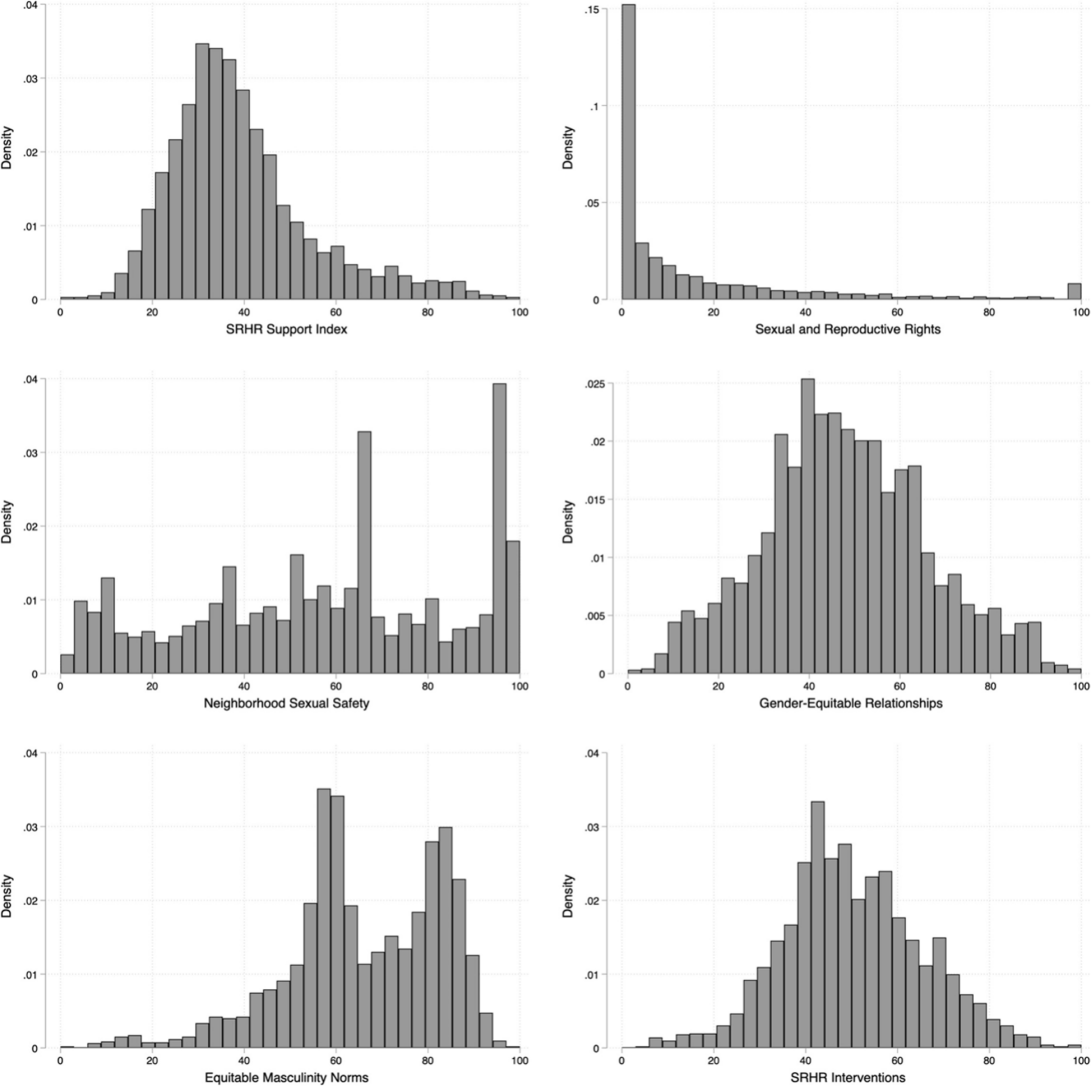
Histograms of the SRHR Support Index and its five subindices in Ethiopia, Kenya, and Zimbabwe (*n* = 3,135)

Figure 1 and Table 3 display the mean and median scores for the full SRHR Support Index and each subindex ranging from 0–100. Each item was coded in such a way that higher values reflect greater support for SRHR according to the Guttmacher-Lancet commission definition [2]). The mean value of the full Index was 39.2 (SD = 15.3) and the median was 36.2 (IQR = 28.9, 46.0), with no significant difference by sex. The lowest scores were found for respondents in Zimbabwe (mean = 32.6, SD = 13.8), followed by Ethiopia (mean = 40.4, SD = 13.6), and the highest in Kenya (mean = 45.5, SD = 13.6).

**Table 3:** Summary statistics of the SRHR Support Index and its five subindices

Population	Mean	Std. Dev	Median	IQR
**SRHR Support Index (Cronbach’s α = 0.80)**				
*Total population*	39.19	15.27	36.22	28.93;46.04
*Ethiopia*	40.41	13.63	37.93	32.94;43.74
*Kenya*	45.48	16.78	43.54	33.20;55.98
*Zimbabwe*	32.65	13.77	30.06	23.72;37.38
*Men*	39.24	15.72	36.27	28.97;46.02
*Women*	39.12	15.76	36.18	28.82;45.92
**Subindex 1: Sexual and Reproductive Rights (Cronbach’s α = 0.92)**				
*Total population*	15.75	23.56	4.67	0.89;20.76
*Ethiopia*	9.95	22.69	1.01	0.71;7.34
*Kenya*	26.38	22.82	21.22	7.38;41.05
*Zimbabwe*	11.13	21.69	1.27	0.86;9.51
*Men*	16.38	23.36	5.47	0.90;22.71
*Women*	15.06	23.73	3.87	0.87;18.38
**Subindex 2: Neighborhood Sexual Safety (Cronbach’s α = 0.86)**				
*Total population*	57.30	28.76	59.45	35.88;81.48
*Ethiopia*	76.10	23.83	85.29	62.39;96.58
*Kenya*	52.59	27.13	56.16	31.54;70.86
*Zimbabwe*	46.09	26.19	46.38	27.01;65.28
*Men*	57.76	28.16	59.42	36.43;81.71
*Women*	56.82	29.38	59.47	34.86;81.20
**Subindex 3: Gender-Equitable Relationship (Cronbach’s α = 0.71)**				
*Total population*	48.01	18.20	47.03	35.71;60.22
*Ethiopia*	49.36	19.11	48.29	36.46;61.45
*Kenya*	55.33	16.81	55.91	43.90;66.07
*Zimbabwe*	40.47	15.54	39.82	32.01;49.54
*Men*	47.56	18.34	46.47	35.35;59.62
*Women*	48.48	18.05	47.43	36.17;60.80
**Subindex 4: Equitable Masculinity Norms (Cronbach’s α = 0.65) **				
*Total population*	65.97	16.93	64.81	56.34;81.01
*Ethiopia*	68.68	16.47	70.39	58.19;82.96
*Kenya*	63.10	18.45	63.04	53.17;79.06
*Zimbabwe*	66.28	15.46	63.49	56.65;80.26
*Men*	64.56	17.22	62.98	55.41;80.02
*Women*	67.47	16.48	67.85	57.41;81.89
**Subindex 5: SRHR Interventions (Cronbach’s α = 0.57)**				
*Total population*	50.26	15.28	48.79	40.41;60.00
*Ethiopia*	51.03	14.86	51.76	41.48;60.74
*Kenya*	53.25	18.10	53.12	39.91;66.82
*Zimbabwe*	47.01	11.95	45.88	40.06;54.36
*Men*	50.97	15.38	49.81	40.98;60.47
*Women*	49.53	15.15	48.05	39.62;59.34

*N* = 3,135. The SRHR Support Index and five subindices are measured on a 0–100 scale

Turning to the subindices, the total mean score was highest for Equitable Masculinity Norms (subindex 4) at 66.0 (SD = 16.9) – indicating relatively high disagreement with stereotypical views on men’s dominance and power, followed by Neighborhood Sexual Safety (subindex 2), SRHR Interventions (subindex 5) at 57.3 (SD = 28.8), and Gender-Equitable Relationships (subindex 3) at 48.0 (SD = 18.2). The lowest overall support was found for Sexual and Reproductive Rights (subindex 1) with mean value 15.7 (SD = 23.6).

Like in the full Index, respondents in Ethiopia and Kenya scored higher on subindex 2, 3 and 5 than those in Zimbabwe. Kenyan respondents also scored higher on subindex 1 than those from other countries, indicating more support for sexual and reproductive rights in this context. There were no notable country differences for subindex 4, nor were there any significant differences in mean scores between men and women on any of the five subindices.

### Sociodemographic characteristics associated with support for SRHR

Table 4 shows results from bivariate and adjusted linear regression models of sociodemographic characteristics and the SRHR Support Index.

**Table 4:** Bivariate and adjusted linear regression models of sociodemographic factors and the SRHR Support Index

	**Model 1**	**Model 2**	**Model 3**	**Model 4**	**Model 5**	**Model 6**
	B (SE)	B (SE)	B (SE)	B (SE)	B (SE)	B (SE)
**Age groups (ref. = 30–39)**						
18–24	0.22 (0.15)					-0.14 (0.16)
25–29	0.52^**^ (0.16)					0.20 (0.16)
40–49	-0.23 (0.18)					-0.11 (0.18)
50–99	-0.84^***^ (0.18)					-0.63^***^ (0.18)
**Sex (ref. = male)**						
Female		-0.04 (0.11)				0.11 (0.11)
**Type of place of residence (ref. = urban)**						
Rural			-0.61^***^ (0.11)			-0.33^**^ (0.11)
**Education (ref. = secondary)**						
Primary or lower						-0.25^*^ (0.12)
Tertiary				0.91^***^ (0.16)		0.81^***^ (0.16)
**Relationship status (ref. = married or cohabiting)**						
Divorced, separated, or widowed					-0.25 (0.18)	-0.05 (0.19)
Single					0.86^***^ (0.12)	0.62^***^ (0.14)
**Constant**	12.54^***^ (0.11)	12.57^***^ (0.07)	12.93^***^ (0.09)	12.65^***^ (0.09)	12.32^***^ (0.07)	12.61^***^ (0.16)
**Observations**	3,113	3,113	3,113	3,113	3,113	3,113
**R-squared**	0.02	-0.00	0.01	0.03	0.02	0.05

^*^p < 0.05, ^**^p < 0.01, ^***^p < 0.001, B = coefficient, SE = standard error, ref. = reference. Table 4 shows results from bivariate (Models 1–5) as well as adjusted (Model 6) regression models of sociodemographic factors and the SRHR Support Index

In bivariate analysis (Models 1–5), the second to the youngest age group (25–29 years) was positively associated with more supportive attitudes towards SRHR (i.e., higher scores), whereas being aged 40 or above was associated with less support, compared to ages 30–39. Higher support for SRHR was also more common among respondents who were single, had higher education, residing in urban areas, compared to the reference groups. There were no significant differences by sex.

In the multivariable model (Model 6), the coefficients for urbanicity, education, and relationship status were attenuated compared to the bivariate models but remained statistically significant with SRHR Support and in the same direction. For age, only the oldest age group (50 + years) was associated with less support for SRHR compared to those aged 30–39 years.

Table 5 further displays adjusted regression findings by country (Models 7–9) and sex (Models 10–11). When stratifying by country, there were no statistically significant differences in SRHR Support Index scores by age, sex, or place of residence. The same patterns with regards to education level and being single were found for Kenya and Zimbabwe as for the total sample across countries, but these associations did not hold in Ethiopia.

**Table 5:** Adjusted linear regression models of the SRHR Support Index in relation to sociodemographic factors, by country and sex

	**Model 7: Ethiopia**	**Model 8: Kenya**	**Model 9: Zimbabwe**	**Model 10: Men**	**Model 11: Women**
	B (SE)	B (SE)	B (SE)	B (SE)	B (SE)
**Age groups (ref. = 30–39)**					
18–24	0.38 (0.25)	-0.47 (0.30)	-0.26 (0.27)	-0.17 (0.26)	-0.11 (0.22)
25–29	0.38 (0.26)	0.06 (0.28)	-0.16 (0.27)	0.15 (0.24)	0.24 (0.23)
40–49	-0.09 (0.30)	-0.05 (0.35)	0.27 (0.25)	-0.11 (0.24)	-0.17 (0.26)
50–99	-0.27 (0.33)	-0.17 (0.46)	0.13 (0.23)	-0.79** (0.24)	-0.45 (0.27)
**Sex (ref. = male)**					
Female	0.32 (0.18)	-0.14 (0.20)	0.18		
**Type of place of residence (ref. = urban)**					
Rural	-0.00 (0.20)	-0.23 (0.20)	-0.26 (0.17)	-0.54*** (0.16)	-0.11 (0.16)
**Education (ref. = secondary)**					
Primary or lower	0.12 (0.22)	-0.35 (0.24)	-0.29 (0.17)	-0.26 (0.17)	-0.18 (0.18)
Tertiary	-0.13 (0.27)	0.72** (0.25)	0.98** (0.31)	0.78*** (0.20)	0.83*** (0.25)
**Relationship status (ref. = married or cohabiting)**					
Divorced, separated, or widowed	-0.30 (0.35)	0.42 (0.39)	-0.07 (0.23)	0.51 (0.34)	-0.29 (0.23)
Single	-0.05 (0.23)	0.73** (0.24)	0.76** (0.25)	0.49* (0.21)	0.82*** (0.21)
**Constant**	12.46*** (0.28)	13.64*** (0.28)	11.33*** (0.23)	12.81*** (0.21)	12.50*** (0.21)
**Observations**	945	1,011	1,157	1,595	1,518
**R-squared**	0.00	0.03	0.02	0.05	0.04

^*^p < 0.05, ^**^p < 0.01, ^***^p < 0.001, B = coefficient, SE = standard error, ref. = reference. Table 5 displays adjusted regression findings by country (Models 7–9) and sex (Models 10–11)

The associations with age and place of residence noted above turned out to be driven by male respondents since no significant relationship was found among females for these variables. Both males and females scored higher on the Index if they had tertiary education and if they were single; these associations were stronger among female respondents.

## Discussion

We aimed to develop a comprehensive index to measure support for SRHR based on nationally representative data collected by WVS in three SSA countries, for which such information has previously been missing. We identified five subindices, reflecting support for: 1) Sexual and Reproductive Rights, 2) Neighborhood Sexual Safety, 3) Gender-Equitable Relationships, 4) Equitable Masculinity Norms, and 5) SRHR Interventions. These five subindices performed well across all three countries and sociodemographic subgroups and were combined into an overall 23-item SRHR Support Index.

Our proposed SRHR Support Index touches upon several aspects of SRHR as defined by the Guttmacher-Lancet framework, such as non-discrimination related to sexuality, sexual orientation and gender identity, reproductive empowerment, consensual and non-violent relationships, satisfying sexual life, gender-equitable relationships, and SRHR information [2]. As such, this Index covers broad aspects of the human rights of all individuals to freely decide on matters related to sexuality and reproduction.

An important contribution of our measure is that it draws on nationally representative samples of both women and men, of all ages and different relationship status, allowing for a more comprehensive understanding of variations in SRHR support. The only previous measure tapping into SRHR attitudes based on WVS data is the three-item Choice subindex [24], which in our study was included in the seven-item subindex “Sexual and Reproductive Rights” capturing the justification of additional SRHR aspects which can be morally, legally, and socially stigmatized or sensitive in many contexts. Since these seven items are included in the core WVS questionnaire, this subindex is readily available for comparative and trend analyses.

In addition, the subindex “Equitable Masculinity Norms” includes three of the 15 items from the Man Box Scale, previously validated in Australia, Mexico, and the United States, indicating that these items can be used in other global settings beyond SSA [25].

While the SRHR Support Index overlaps conceptually with existing indices and scales that capture attitudes towards specific dimensions of SRHR – such as gender equality and women’s empowerment (e.g., SWPER Index [23], Women and Girls Sexual and Reproductive Empowerment Index (WGE-SRH) [26], Women’s Agency Scale [27], the Gender-Equitable Men Scale [28], the G-NORM scale [29]) or fertility (e.g., the Fertility Norms Scale [30]) – its comprehensive approach can help to further improve the understanding and tracking of countries’ progress towards realizing SRHR for all. This Index can in particular be useful to understand and advance SRHR in light of global backlashes and resistance towards sexual and reproductive rights (e.g., abortion and the rights and freedoms of sexual minorities). By focusing on supportive attitudes, the Index or its sub-components can give an indication of developments toward a more favorable landscape for advancing SRHR based on the degree to which individuals and groups support different aspects.

In terms of sociodemographic variations, being single or highly educated was associated with more support for SRHR, except in Ethiopia. Similarly, analyses of global WVS data have found supportive attitudes towards homosexuality, divorce, and abortion (as captured via the Choice index) to be closely linked with higher education, postponed marriage, delayed childbearing, and reduced number of children [24]. While there were no overall differences by sex, younger age and urban residence were associated with more supportive attitudes among males, but not females. The fact that younger male respondents had more supportive attitudes towards SRHR may indicate a generational shift or life-course differences, particularly in urban settings also seen in another study from Kenya [31].

The limitations of the current study should also be considered. First, the index development was inductively driven by data, rather than deductively by the Guttmacher-Lancet framework. Consequently, some components of SRHR were covered to a lesser extent, for example, HIV/AIDS, antenatal care, satisfying sexual life, or sexual orientations besides homosexuality [2]. In addition, the analysis was restricted to available WVS data in Ethiopia, Kenya, and Zimbabwe, limiting the generalizability of findings within and outside of SSA where further validation is needed [32]. The robust performance of the Index across the three countries in the current study is nonetheless a key strength. Its association with several sociodemographic factors (e.g., sex, age, education, and relationship status), previously shown to drive attitudes towards gender and sexuality [15, 21, 33, 34], further supports the validity of the measure. Since we first developed this index, 9 items from the SRHR module have been integrated into the core questionnaire of WVS wave 8, due to be rolled out in 2024-2025, allowing for global comparisons. Four of these are found in our sub-indices 4) Gender-Equitable Relationships and 5) SRHR Interventions. Finally, our ambition is to further validate the proposed Index and subindices in additional contexts, such as India (favorably, using confirmatory instead of exploratory factor analysis) as new data becomes available.

## Conclusion

This study addresses two important gaps in the existing literature: the need for new comprehensive measurements of support for SRHR, and the widespread data gap on such perceptions in SSA. The comprehensive SRHR Support Index proposed in this paper has the potential to broaden research on the extent to which individuals and groups support SRHR, which is highly relevant given how SRHR are becoming increasingly contested worldwide. The index performed well across countries and sociodemographic subgroups, but further validation is needed to assess its applicability in different settings and populations. This could be done by integrating all items from the Index and its subindices into the standard WVS module (covering 100 + countries) as well as other global surveys, thereby providing a baseline against which to track development over time. Doing so would also allow for broadening research on the influence of attitudes and norms on related SRHR outcomes, by linking data on the perceptions of individuals and groups (e.g., from the WVS) with health, social, and development outcomes (e.g., from the DHS). As we approach 2030, robust measurements of support and resistance towards individuals’ rights to decide over their own bodies, sexuality, and reproduction is critical to track progress towards achieving SRHR for all as part of the SDGs. The SRHR Support Index offers a tool to further advance our understanding of attitudes and norms as barriers or facilitators to SRHR globally, thereby guiding the tailoring of interventions as well as policy.

### Supplementary Information

Supplementary Material 1. 

## Data Availability

The data used in this study are publicly available from https://www.worldvaluessurvey.org/. STATA do-files can be requested from the corresponding author.

## Abbreviations

DHS: Demographic and Health Surveys
EFA: Exploratory Factor Analysis
GEAS: The Global Early Adolescent Study
HIV: Human Immunodeficiency Virus
IPF: Iterated Principal Factors
KMO: Kaiser-Meyer-Olkin
ML: Maximum Likelihood
PA: Parallel Analysis
PCA: Principal Component Analysis
SE: Standard Error
SD: Standard Deviation
SDG: Sustainable Development Goals
SRHR: Sexual and Reproductive Health and Rights
SSA: Sub-Saharan Africa
WVS: World Values Survey
