# Supplementary material for: Support for sexual and reproductive health and rights in Sub-Saharan Africa: a new index based on World Values Survey data
Source: Reprod Health. 2024 Jun 25;21:90. doi: 10.1186/s12978-024-01820-2 (PMC11197335; doi:10.1186/s12978-024-01820-2)
Supplement: Supplementary file 1 — Supplementary Material 1. [file 12978_2024_1820_MOESM1_ESM.docx]

## SUPPLEMENTARY MATERIALS

**Table A1** Key indicators for SRHR and gender equality in Ethiopia, Kenya, Zimbabwe, and the World as a comparison

| **Indicator** | **World** | **Ethiopia** | **Kenya** | **Zimbabwe** |
| --- | --- | --- | --- | --- |
| Population, million (UNFPA, 2022) | 7 954 | 120.8 | 56.2 | 15.3 |
| Life expectancy at birth female/male (UNFPA, 2022) | 76 / 71 | 70 / 66 | 70 / 65 | 64 / 60 |
| Total Fertility Rate (UNFPA, 2022) | 2.4 | 3.9 | 3.3 | 3.3 |
| Population with at least some secondary education, % of ages 25 and older, female/male (UNDP, 2021) (SDG4.4) | 64.2 / 70.3 | 9.1 / 20.1 | 31.1 / 37.7 | 61.8 / 72.4 |
| Share of seats in parliament, % held by women (UNDP, 2021) (SDG5.5) | 25.9 | 39.5 | 23.2 | 34.6 |
| Gender inequality index (GII) rank out of 170 countries (UNDP, 2021) | - | 129 | 128 | 134 |
| Poverty headcount ratio at $2.15 a day (2017 PPP) (% of population)  (World Bank) | 8.4  (2017) | 27  (2015) | 29.4  (2015) | 39.8  (2019) |
| Maternal Mortality Ratio per 100.000 live births (UNFPA, 2017) (SDG3.1) | 211 | 401 | 342 | 458 |
| Contraceptive prevalence rate modern methods, all women & married/in union aged 15–49 (UNFPA, 2022) | 44% all  57% married | 30% all  42% married | 43% all  59% married | 49% all  69% married |
| Adolescent birth rate, births per 1,000 girls/women aged 15-19 (UNDP, 2021) (SDG3.7) | 42.5 | 69.2 | 64.2 | 94.3 |
| Child marriage <18 years old (UNFPA, 2005–2020) | 26% | 40% | 23% | 34% |
| FGM girls aged 15–19 (UNFPA, 2004–2020) | - | 65% | 21% | - |
| Intimate partner violence, past 12 months (UNFPA 2018) | 13% | 27% | 23% | 18% |
| Decision making on sexual and reproductive health and reproductive rights (UNFPA, 2007–2020) | 57% | 45% | 56% | 60% |
| Grounds for right to abortion  (Center for Reproductive Rights, 2020) | - | Broad social or economic grounds | Preserve Health | Preserve Health |
| Criminalization of consensual same-sex sexual acts between adults (ILGA, 2020) | - | YES Up to 8 years prison | YES 10 years to life in prison | YES Up to 8 years prison |

Sources: <https://www.unfpa.org/data/world-population/ET>

<https://www.unfpa.org/data/world-population/KE>

<https://www.unfpa.org/data/world-population/NG>

<https://www.unfpa.org/data/world-population/ZW>

World Bank data: <https://data.worldbank.org/>

UNFPA: <https://www.unfpa.org/data/world-population-dashboard>

UNDP: <https://hdr.undp.org/data-center/documentation-and-downloads>

Center for Reproductive Rights: <https://reproductiverights.org/worldabortionlaws>

ILGA: <https://ilga.org/maps-sexual-orientation-laws>

| **Table A1** Overview of WVS survey items included in the analysis along with response options | |
| --- | --- |
| **Item** | **Response options** |
| Justifiable: To use contraceptives | 10-graded scale always-never justified |
| Justifiable: Female genital circumcision | 10-graded scale always-never justified |
| Justifiable: Prostitution | 10-graded scale always-never justified |
| Justifiable: For a man to beat his wife | 10-graded scale always-never justified |
| Justifiable: Parents beating children | 10-graded scale always-never justified |
| Justifiable: Sex before marriage | 10-graded scale always-never justified |
| Justifiable: Having casual sex | 10-graded scale always-never justified |
| Justifiable: Divorce | 10-graded scale always-never justified |
| Justifiable: Homosexuality | 10-graded scale always-never justified |
| Justifiable: Abortion | 10-graded scale always-never justified |
| Honor-related oppression deals with the fact that the family or relatives decide primarily on young women’s life choices. What is your opinion? | 10-graded scale can never be accepted–should always be accepted |
| Only when a woman has a child is she a real woman | Strongly agree, agree, disagree, or strongly disagree |
| Having a son is always better than having a daughter | Strongly agree, agree, disagree, or strongly disagree |
| It’s a woman’s responsibility to avoid getting pregnant | Strongly agree, agree, disagree, or strongly disagree |
| A man should use violence, if necessary, to get respect | Strongly agree, agree, disagree, or strongly disagree |
| A man shouldn't have to do household chores | Strongly agree, agree, disagree, or strongly disagree |
| It is acceptable to have sex with someone in exchange for gifts and favors | Strongly agree, agree, disagree, or strongly disagree |
| People who dress, act or identify as the opposite sex should be treated just as anyone else | Strongly agree, agree, disagree, or strongly disagree |
| A "real man" should have as many sexual partners as he can | Strongly agree, agree, disagree, or strongly disagree |
| A man who talks a lot about his worries, fears, and problems doesn’t deserve respect | Strongly agree, agree, disagree, or strongly disagree |
| A homosexual guy is not a "real man" | Strongly agree, agree, disagree, or strongly disagree |
| A man who cannot father children is not a real man | Strongly agree, agree, disagree, or strongly disagree |
| Sexuality education helps people make informed decisions | Strongly agree, agree, disagree, or strongly disagree |
| Women should have access to safe abortion services (to terminate an unwanted pregnancy) | Strongly agree, agree, disagree, or strongly disagree |
| A couple who wants to have children but cannot conceive should have access to infertility services | Strongly agree, agree, disagree, or strongly disagree |
| Even if a girl does not want to be married, she should honor the decisions/wishes of her family | Strongly agree, agree, disagree, or strongly disagree |
| Even if a boy does not want to be married, he should honor the decisions/wishes of his family | Strongly agree, agree, disagree, or strongly disagree |
| A girl should wait to have children until she is at least 18 years old, even if she is married | Strongly agree, agree, disagree, or strongly disagree |
| It is safer for a woman to give birth at a clinic than at home | Strongly agree, agree, disagree, or strongly disagree |
| A girl is ready for marriage once she starts menstruating | Strongly agree, agree, disagree, or strongly disagree |
| Contraceptives should be available for everyone, whether or not one is married | Strongly agree, agree, disagree, or strongly disagree |
| If a man has a girlfriend or wife, he should know where she is all the time | Strongly agree, agree, disagree, or strongly disagree |
| No-one else should interfere when it comes to violence within a family | Strongly agree, agree, disagree, or strongly disagree |
| There is no doubt that gainful employment is good but that what most women really want is a home and children | Strongly agree, agree, disagree, or strongly disagree |
| A woman should tolerate violence to keep the family together | Strongly agree, agree, disagree, or strongly disagree |
| A man should always have the final say about decisions in his relationship or marriage | Strongly agree, agree, disagree, or strongly disagree |
| On the whole, family life suffers when women work full time | Strongly agree, agree, disagree, or strongly disagree |
| It is a man’s job to earn money and a women’s job to take care of home and family | Strongly agree, agree, disagree, or strongly disagree |
| Sexuality education promotes sexual activity among young people | Strongly agree, agree, disagree, or strongly disagree |
| A woman who shows that she is interested in sex is considered indecent/rude/illmannered/vulgar | Strongly agree, agree, disagree, or strongly disagree |
| A husband shouldn’t have to do household chores | Strongly agree, agree, disagree, or strongly disagree |
| A man who discusses important decisions with his wife is considered weak | Strongly agree, agree, disagree, or strongly disagree |
| A man should pay more attention to his mother's opinion than his wife's | Strongly agree, agree, disagree, or strongly disagree |
| Men may use violence to keep their wives in line | Strongly agree, agree, disagree, or strongly disagree |
| Parents may use violence or threats when bringing up their children | Strongly agree, agree, disagree, or strongly disagree |
| It is a man’s duty to exercise guardianship over his female relatives | Strongly agree, agree, disagree, or strongly disagree |
| It is not good for a boy to be taught how to cook, sew, clean the house, and take care of younger children | Strongly agree, agree, disagree, or strongly disagree |
| Men should really be the ones to bring money home to provide for their families, not women | Strongly agree, agree, disagree, or strongly disagree |
| When a mother works for pay, the children suffer | Strongly agree, agree, disagree, or strongly disagree |
| On the whole, men make better political leaders than women | Strongly agree, agree, disagree, or strongly disagree |
| A university education is more important for a boy than a girl | Strongly agree, agree, disagree, or strongly disagree |
| On the whole, men make better business leaders than women do | Strongly agree, agree, disagree, or strongly disagree |
| Being a housewife is just as fulfilling as working for pay | Strongly agree, agree, disagree, or strongly disagree |
| Homosexual couples are as good parents as other couples | Strongly agree, agree, neither agree nor disagree, disagree, or strongly disagree |
| How frequently do the following occur in your neighborhood: Sexual assault/rape | Very frequently, quite frequently, not frequently, or not at all |
| How frequently do the following occur in your neighborhood: Men and boys hurting women and girl | Very frequently, quite frequently, not frequently, or not at all |
| How frequently do the following occur in your neighborhood: Men and boys making unwanted sexual comments or gestures toward girls or women | Very frequently, quite frequently, not frequently, or not at all |
| How frequently do the following occur in your neighborhood: Women and girls trading sex for money | Very frequently, quite frequently, not frequently, or not at all |

| **Table A3** Final five-factor solution with loadings for each of the 23 variables in the SRHR Support Index | | | | | | | | |
| --- | --- | --- | --- | --- | --- | --- | --- | --- |
| **Variable** | **Factor 1** | **Factor 2** | **Factor 3** | **Factor 4** | **Factor 5** | **Unique-ness** | **Variable names** | **Comment** |
| **FACTOR 1: “Sexual and Reproductive Rights”** | | | | | | | | |
| Q182 | 0.8557 | 0.0359 | -0.0332 | 0.0007 | -0.0779 | 0.2676 | Justifiable: Homosexuality | * |
| Q183 | 0.8838 | 0.0096 | -0.0263 | 0.0472 | -0.0453 | 0.2275 | Justifiable: Prostitution |  |
| Q184 | 0.8413 | 0.0283 | 0.0346 | 0.0112 | 0.0532 | 0.2834 | Justifiable: Abortion | * |
| Q185 | 0.6642 | 0.0011 | 0.0390 | 0.0687 | 0.1005 | 0.5383 | Justifiable: Divorce | * |
| Q186 | 0.7625 | -0.0215 | 0.0271 | -0.0249 | 0.0806 | 0.4012 | Justifiable: Sex before marriage |  |
| Q189 | -0.7236 | 0.0298 | 0.0782 | 0.0361 | 0.0796 | 0.4614 | Justifiable: For a man to beat his wife |  |
| Q193 | 0.8295 | -0.0069 | 0.0096 | -0.0301 | 0.0041 | 0.3055 | Justifiable: Having casual sex |  |
| **FACTOR 2: “Neighborhood Sexual Safety”** | | | | | | | | |
| H311 | 0.0114 | 0.7112 | -0.0481 | 0.0839 | 0.0234 | 0.4848 | How often in neighborhood: Sexual assault/rape |  |
| H312 | 0.0190 | 0.8347 | 0.0597 | -0.0249 | -0.0017 | 0.2949 | How often in neighborhood: Women and girls trading sex for money |  |
| H313 | -0.0045 | 0.9159 | -0.0082 | 0.0417 | 0.0300 | 0.1554 | How often in neighborhood: Men and boys hurting women and girls |  |
| H314 | 0.0175 | 0.8731 | -0.0151 | -0.0784 | -0.0767 | 0.2304 | How often in neighborhood: Men and boys making unwanted sexual comments or gestures toward girls or women |  |
| **FACTOR 3: “Gender-Equitable Relationships”** | | | | | | | | |
| H325 | -0.0449 | -0.0553 | 0.5623 | 0.0946 | 0.0048 | 0.6480 | A man should always have the final say about decisions in his relationship or marriage |  |
| H326 | 0.0472 | -0.0743 | 0.5950 | -0.0091 | -0.0434 | 0.6473 | If a man has a girlfriend or wife, he should know where she is all the time | (Z) |
| H328 | -0.0061 | 0.0091 | 0.7346 | -0.0559 | -0.0344 | 0.4808 | Gainful employment is good but home and children is what most women really want |  |
| H329 | 0.1208 | -0.0245 | 0.5770 | 0.0320 | -0.0421 | 0.6402 | On the whole, family life suffers when women works full time | (K) |
| H330 | -0.1046 | 0.0875 | 0.6746 | 0.0213 | 0.0259 | 0.5113 | It is a man's job to earn money and a woman's job to take care of home and family |  |
| **FACTOR 4: “Masculinity norms”** | | | | | | | | |
| H348 | -0.0297 | 0.0507 | 0.1255 | 0.5366 | 0.0443 | 0.6372 | A man who talks a lot about his worries, fears, and problems doesn't reserve respect | ** |
| H350 | -0.0050 | -0.0147 | -0.0704 | 0.7941 | 0.0075 | 0.3921 | A real man should have as many sexual partners as he can | ** |
| H351 | 0.0419 | -0.0093 | 0.0415 | 0.8186 | 0.0044 | 0.3132 | A man should use violence, to get respect, if necessary | ** |
| **FACTOR 5: “Sexual and Reproductive Health and Rights Interventions”** | | | | | | | | |
| H337 | 0.0072 | 0.1008 | 0.1836 | -0.2230 | 0.5261 | 0.6871 | Women should have access to safe abortion services (to terminate an unwanted pregnancy) |  |
| H341 | 0.0224 | -0.0719 | -0.0483 | -0.0650 | 0.5808 | 0.6658 | Contraceptives should be available for everyone, whether or not one is married | (E) |
| H343 | 0.0589 | -0.0643 | -0.0689 | 0.1067 | 0.6184 | 0.5741 | Sexual education helps people make informed decisions |  |
| H344 | -0.1116 | 0.0282 | -0.0645 | 0.1355 | 0.6021 | 0.5793 | A couple who cannot conceive should have access to infertility services |  |
| *Notes*: Variables with low loading (<0.50) and high uniqueness (>0.70) are marked (E) for Ethiopia, (K) for Kenya, and (Z) for Zimbabwe.  *=variable included in WVS Choice Index; **=variable included in Man Box Scale | | | | | | | | |

| **Table A4** Correlation matrix of scores derived from the final five-factor model | | | | | |
| --- | --- | --- | --- | --- | --- |
|  | **Factor 1** | **Factor 2** | **Factor 3** | **Factor 4** | **Factor 5** |
| **Factor 1** | 1 |  |  |  |  |
| **Factor 2** | -0.03 | 1 |  |  |  |
| **Factor 3** | 0.11 | 0.11 | 1 |  |  |
| **Factor 4** | -0.13 | 0.05 | 0.30 | 1 |  |
| **Factor 5** | 0.15 | -0.03 | 0.18 | 0.20 | 1 |

| **Table A5** Sociodemographic characteristics of the full sample (n=3,711) | | |
| --- | --- | --- |
| **Variable** | **n** | **%** |
| **Age** |  |  |
| *18–24* | 1,029 | 27.73 |
| *25–29* | 729 | 19.64 |
| *30–39* | 922 | 24.85 |
| *40–49* | 511 | 13.77 |
| *50–99* | 509 | 13.72 |
| *Missing* | 11 | 0.30 |
| **Sex** |  |  |
| *Man* | 1,859 | 50.09 |
| *Woman* | 1,845 | 49.72 |
| *Missing* | 7 | 0.19 |
| **Place of residence** |  |  |
| *Urban* | 1,410 | 38.00 |
| *Rural* | 2,301 | 62.00 |
| **Relationship status** |  |  |
| *Married or cohabiting* | 2,189 | 58.99 |
| *Divorced, separated, or widowed* | 377 | 10.16 |
| *Single* | 1,138 | 30.67 |
| *Missing* | 7 | 0.19 |
| **Education** |  |  |
| *Primary or lower* | 1,878 | 50.61 |
| *Secondary* | 1,238 | 33.36 |
| *Tertiary* | 581 | 15.66 |
| *Missing* | 14 | 0.38 |
| **Country** |  |  |
| *Ethiopia* | 1,230 | 33.14 |
| *Kenya* | 1,266 | 34.11 |
| *Zimbabwe* | 1,215 | 32.74 |
| **TOTAL** | **3,711** | **100.0** |


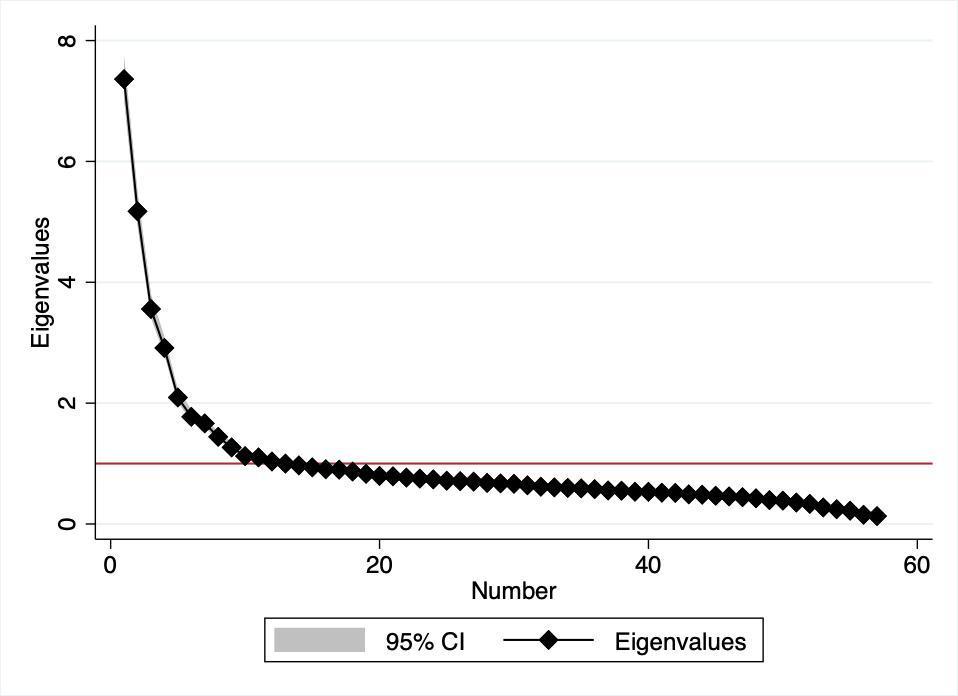


**Figure A1** Scree plot of pooled Exploratory Factor Analysis (EFA) with the initial 58 items (N=2,722)


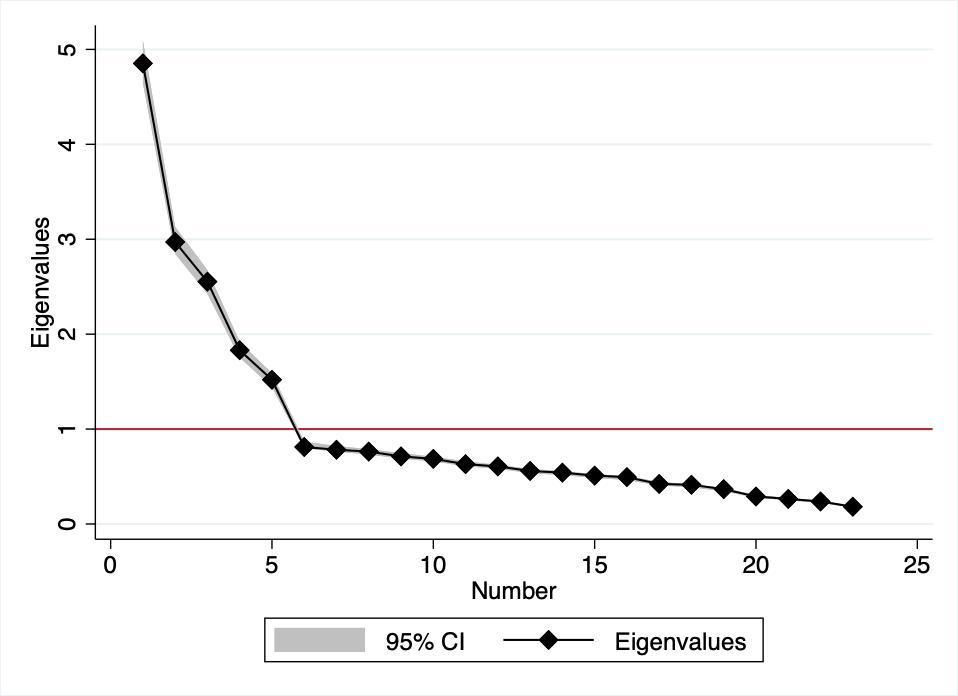


**Figure A2** Scree plot of pooled Exploratory Factor Analysis (EFA) with the retained 23 items for the SRHR Support Index (n=3,135)


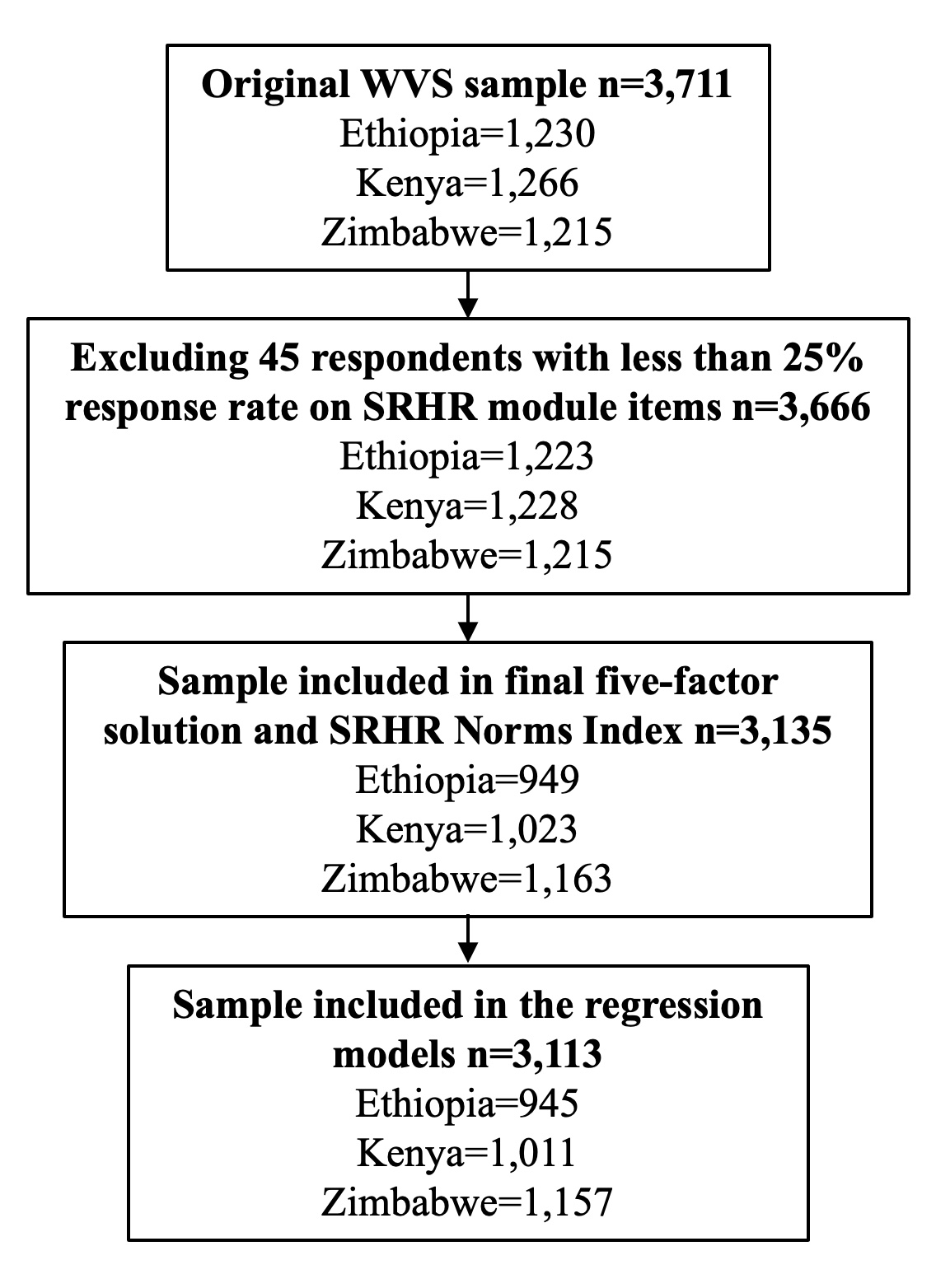


**Figure A3** Sample selection flowchart
